# Management of oral anticoagulant therapy after intracranial hemorrhage in patients with atrial fibrillation

**DOI:** 10.3389/fcvm.2023.1061618

**Published:** 2023-05-25

**Authors:** Fabiana Lucà, Furio Colivicchi, Fabrizio Oliva, Maurizio Abrignani, Giorgio Caretta, Stefania Angela Di Fusco, Simona Giubilato, Stefano Cornara, Concetta Di Nora, Andrea Pozzi, Irene Di Matteo, Anna Pilleri, Carmelo Massimiliano Rao, Antonio Parlavecchio, Roberto Ceravolo, Francesco Antonio Benedetto, Roberta Rossini, Raimondo Calvanese, Sandro Gelsomino, Carmine Riccio, Michele Massimo Gulizia

**Affiliations:** ^1^ Cardiology Department, Grande Ospedale Metropolitano di Reggio Calabria, GOM, Azienda Ospedaliera Bianchi Melacrino Morelli, Italy; ^2^ Cardiology Division, San Filippo Neri Hospital, ASL Roma 1, Roma, Italy; ^3^ De Gasperis Cardio Center, ASST Niguarda Hospital, Milano, Italy; ^4^ Cardiology Unit, Paolo Borsellino Hospital, ASP Trapani, Marsala, Italy; ^5^ Cardiology Unit, Sant'Andrea Hospital, ASL 5 Liguria, La Spezia, Italy; ^6^ Cardiology Division Cannizzaro Hospital, Catania, Italy; ^7^ Cardiology Division San Paolo Hospital, ASL 2, Savona, Italy; ^8^ Cardiology Division, Maria della Misericordia di Udine, Italy; ^9^ Cardiology Division, Valduce Hospital, Como, Italy; ^10^ Cardiology Division, Brotzu Hospital, Cagliari, Italy; ^11^ Cardiology Division, Giovanni Paolo II Hospital, Lamezia Terme, Italy; ^12^ Cardiology Division S. Croce e Carle Hospital, Cuneo, Italy; ^13^ Cardiology Division, Ospedale del Mare, Napoli, Italy; ^14^ Cardiothoracic Department, Maastricht University, Maastricht, The Netherlands; ^15^ Cardiovascular Department, A.O.R.N. Sant'Anna e San Sebastiano, Caserta, Italy; ^16^ Cardiology Department, Garibaldi Nesima Hospital, Catania, Italy

**Keywords:** atrail fibrillation, oral anti coagulation, left atrial appendage (LAA) occlusion, intracranial hemorrhage, NOAC drugs

## Abstract

Intracranial hemorrhage (ICH) is considered a potentially severe complication of oral anticoagulants (OACs) and antiplatelet therapy (APT). Patients with atrial fibrillation (AF) who survived ICH present both an increased ischemic and bleeding risk. Due to its lethality, initiating or reinitiating OACs in ICH survivors with AF is challenging. Since ICH recurrence may be life-threatening, patients who experience an ICH are often not treated with OACs, and thus remain at a higher risk of thromboembolic events. It is worthy of mention that subjects with a recent ICH and AF have been scarcely enrolled in randomized controlled trials (RCTs) on ischemic stroke risk management in AF. Nevertheless, in observational studies, stroke incidence and mortality of patients with AF who survived ICH had been shown to be significantly reduced among those treated with OACs. However, the risk of hemorrhagic events, including recurrent ICH, was not necessarily increased, especially in patients with post-traumatic ICH. The optimal timing of anticoagulation initiation or restarting after an ICH in AF patients is also largely debated. Finally, the left atrial appendage occlusion option should be evaluated in AF patients with a very high risk of recurrent ICH. Overall, an interdisciplinary unit consisting of cardiologists, neurologists, neuroradiologists, neurosurgeons, patients, and their families should be involved in management decisions. According to available evidence, this review outlines the most appropriate anticoagulation strategies after an ICH that should be adopted to treat this neglected subset of patients.

## 1. Introduction

Intracranial Hemorrhage (ICH) is a well-recognized complication of oral anticoagulants (OACs) and antiplatelet therapy (APT) (1, 2). In patients with atrial fibrillation (AF) treated with OACs who survived a previous ICH, the risk of recurrent bleeding ranges from 1.3% to 7.4% (3, 4). It has been estimated that an annual rate of ICH of 0.3%–0.6% and 0.1%–0.2% in patients receiving vitamin K antagonists (VKA) and Direct oral anticoagulants (DOACs), respectively (5–7).

Therefore, OACs therapy in ICH survivors with AF is a clinical challenge. The risk of recurrent ICH might be balanced against the thromboembolic risk related to AF if patients are not adequately treated (8).

Although clinicians often encounter this issue in clinical practice, patients with a recent ICH and AF are poorly or not represented in randomized controlled trials (RCTs) on ischemic stroke risk management in AF (9).

Furthermore, the limited data available on whether AF patients surviving an ICH may benefit from OACs are conflicting (10).

It would be advisable to carefully consider the presence of specific risk factors, as well as the causes of ICH and neuroimaging findings in deciding whether to restart or initiate an OACs regimen after an ICH.

Finally, left atrial appendage (LAA) occlusion should be evaluated in AF patients with a high risk of recurrent ICH (11). This systematic review aims to summarize ICH's nosology and epidemiology and report available evidence on anticoagulation management after ICH in patients with AF. We also outline and discuss the most appropriate therapeutic strategies in patients with AF who have survived ICH, as national and international experts suggested.

## 2. Materials and methods

### 2.1. Search strategy

Literature sources have been investigated according to the rules of Preferred Reporting Items for Systematic Reviews and Meta-Analyses (PRISMA) (12).

Three authors (CD, IDM, AP) established the search strategy, which was then approved by another author (FL). PubMed, Medline, and SCOPUS databases have been used. The search was performed including the following terms (novel oral anticoagulants OR “NOACs” OR direct-acting oral anticoagulants OR DOACs OR anticoagulant drugs OR Vitamin K antagonists OR VKA) AND “atrial fibrillation” AND (Hemorrhagic stroke OR intracerebral hemorrhages) AND percutaneous left atrial appendage closure. Titles and abstracts of all articles published between January 2005 and March 2022 were initially analyzed. The literature was restricted to articles published in English. Three expert investigators (FC, SG, MG) performed queries and identified articles. The PRISMA checklist is reported in the Supplemental Material. The protocol has been registered on Prospero (n° 350413).

Additional articles found as references in original papers were crosschecked for inclusion.

### 2.2. Selection criteria and quality assessment

RCTs have not been included in the analysis. The inclusion criteria of the studies are listed below:

(1) human studies; (2) full articles about AF and ICH; (3) adequate information reported about the patient's assessment. Exclusion criteria were: (1) experimental animal (2) case reports, reviews, and (5) partial information for meta-analysis.

Two authors (FL and SADF) were responsible for selecting and extracting the studies and information, and outcomes of patients. Two reviewers (S.G. and MMG) independently evaluated the eligibility.

### 2.3. Results

**Figure 1:**
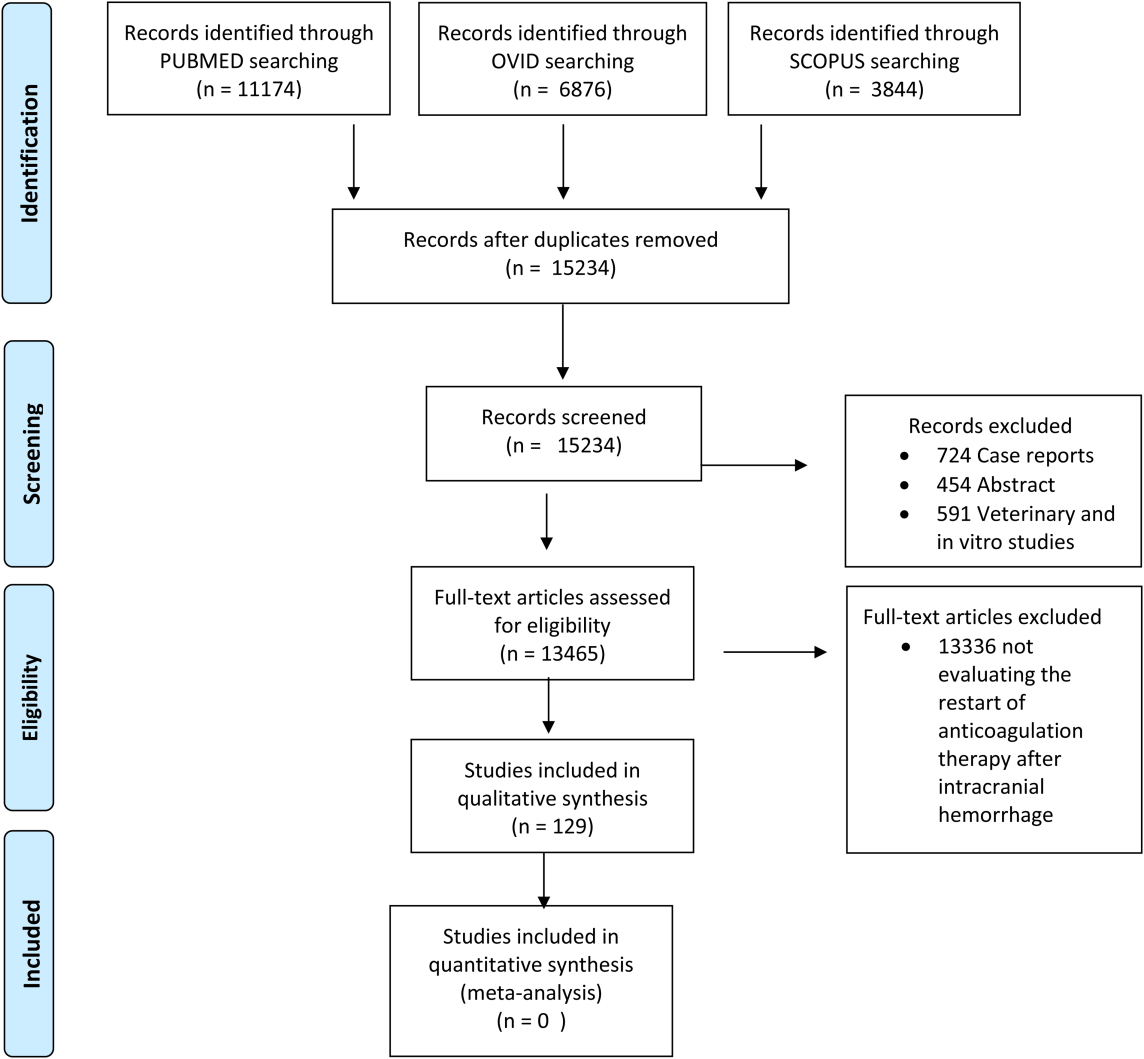
Prisma flow diagram.

The PRISMA flow diagram reporting the study selection process and exclusion reasons are represented in Figure 1. The number of studies screened was 15,234.

Of these articles, 724 have been excluded as case reports, 454 as abstracts, and 591 as animal and experimental studies. Therefore, 13,465 full-text articles underwent further screening after the exclusion of 13,336 results which were not considered to be on-topic and suitable for other evaluation. Subsequently, 129 articles were further assessed.

## 3. Discussion

### 3.1. ICH nosology and etiology

ICH accounts for 10%–20% of all strokes and is correlated to higher mortality and a worse functional outcome (13). Three types of ICH have been described: (1) epidural hemorrhage (EDH)/subdural hemorrhage (SDH), (2) subarachnoid hemorrhage (SAH), (3) and intraparenchymal hemorrhage (IPH)/intraventricular hemorrhage (IVH) (14).

ICH is classified conventionally as primary or secondary based on the leading causes. A primary ICH due to hypertensive injury or cerebral amyloid angiopathy (CAA) damage, resulting in the spontaneous rupture of small vessels, has been reported in 80% of cases (15).

In this regard, cerebral vasculopathy due to chronic hypertension has been described to be the leading cause of ICH (16).

The small, penetrating arteries arising from the anterior, middle, or posterior cerebral arteries and from basilar branches have been reported as the most common site of hypertensive ICH (15). Conversely, it has been well-assessed that CAA is the leading cause of ICH in elderly people (17).

What in the classification of ICH should be considered most is its location in order to distinguish lobar or non-lobar and supratentorial or infratentorial (18). It has been shown that CAA is a common cause of lobar ICH (19). The rupture of cortical medium and small arterioles is frequently due to beta-amyloid deposition, causing both asymptomatic microhemorrhages and symptomatic lobar hemorrhages (20).

Basal ganglia, thalamus, pons, cerebellum, and the subcortical white matter have been described as the most common sites involved with hypertensive ICH characterized by the lipohyalinosis of small perforating arteries occurrence (21).

On the contrary arteriovenous malformations, cavernous angiomas, cerebral aneurysms, and aortic-venous fistulae, neoplasms, hemorrhagic conversion of an ischemic stroke, vasculitis, drug abuse, and bleeding diathesis have been reported as congenital and acquired causes of secondary ICH (22).

SAH, which is less common, occurs when bleeding takes place between the inner and outer layers of the tissue surrounding the brain.

A spontaneous occurrence has been described. However, head trauma could also result in SAH (23). Spontaneous (primary) SAH usually results from ruptured intracranial aneurysms. A congenital saccular or berry aneurysm is the cause of SAH in about 85% of patients (24). Aneurysmal hemorrhage may occur at any age ranging mainly from ages 40 to 65 (23). Brain aneurysms have been associated with smoking, female sex, and high blood pressure. Less common causes are arteriovenous malformations, mycotic aneurysms, bleeding disorders, and the use of blood thinners (23, 24).

### 3.2. Epidemiological aspects and risk factors of hemorrhagic stroke

#### 3.2.1. Incidence

ICH has a very variable incidence in relation to geographical areas (it accounts for about 8%–15% of strokes in Western countries and more in developing ones) and ethnic groups (with the greatest incidence among Asians) (25). Its incidence increases with advancing age and is higher in men (25–27). It has also been associated with overweight, tobacco use, and alcohol assumption. Besides, male patients with ICH are younger than female patients (28).

#### 3.2.2. Time trends

Worldwide, the absolute number of ICH cases is increasing, with an increase in incidence in the poorest countries and a reduction in China (29) and Western countries, likely due to better blood pressure control at the population level (25). In the Tromsø Study in Norway, ICH occurrence dropped in women between 1994 and 2013, with a reduction in non-lobar ICH, while incidence rates in men remained stable (IRR: 1.27, 95% CI: 0.69–2.31) (30). In a Danish study, age- and sex-related rates of ICH were inferior in the population cohort ranging between 33% to 28 in the years 2004–2005 and 2016–2017 respectively (31). Among patients aged ≥70 years, a statistically non-significant time trend in hemorrhagic stroke incidence reduction has been observed. However, evidence in incidence trends is conflicting. The Dijon register (27) shows that the incidence of ICH has remained relatively stable from 1985 to 2008 (12.4/100,000/year). In recent data from this study, stroke rates increased over time from 1987 to 2012 independently from the subtype of stroke (32), and the sex gap in incidence remained unchanged (27). In the French national hospital discharge database, from 2008 to 2014, the incidence of subjects referred to the hospital for hemorrhagic stroke was not dependent on age and sex, and it was stable (33). These data can be explained by the aging of the population, in conjunction with increased use of antithrombotic therapy and the increased presence of CAA, whose estimated prevalence in patients with ICH is 14.7% (34).

#### 3.2.3. Risk factors

It has been shown that old age, male sex, Asian ethnicity, and the presence of CAA are non-modifiable risk factors for ICH. Chronic kidney failure (CKD), which is a marker of small cerebral vessel disease (25), is a further condition associated with increased ICH risk. Hypertension (30), smoking, and excessive alcohol consumption have been reported as modifiable factors associated with about 9 out of 10 cases of ICH. A possible role of low-density lipoprotein (LDL) cholesterol and triglycerides levels have been hypothesized, although it has not been demonstrated (25). Arterial hypertension boosts the odds of ICH, with very high blood pressure values (systolic blood pressure > 180 mmHg) at a presentation being an independent predictor of deep localization (34). Moreover, it is more strictly correlated with non-lobar than lobar hemorrhage (35). In Denmark, the prevalence of hypertension in the ICH population rose to 66% in the period between 2004 and 2017, probably because of the absence of an appropriate therapy or a suboptimal strategy in most patients (35). In northeast China, the high number of patients with inadequate pressure control has been correlated with significant stroke incidence (36). Regarding the relationship between alcohol exposure and stroke, an alcohol dehydrogenase genotype 1B (ADH1B, rs1229984), heterozygous or homozygous in drinks consumers, has been associated with an increased ICH risk (37).

Notably, recreational drugs with sympathomimetic action (including cocaine, heroin, and amphetamines) have been associated with increased ICH risk (38). The use of antiplatelet agents, mainly if associated with OACs, also increases ICH risk.

### 3.3. ICH natural history (risk of death or a new stroke, stratification of thrombotic/hemorrhagic risk)

ICH has high morbidity and mortality rates, which at one month is approximately 45% (39).

Although the significant morbidity and mortality have led the scientific community to focus on hemorrhagic stroke management, its prognosis has not improved significantly during the last several decades.

The most important predictors of death are older age, a low score on the Glasgow Coma Scale (40), greater ICH volume, the presence of intraventricular hemorrhage, and deep/infratentorial ICH area (15). Most of these factors are also included in the ICH score, which allows risk estimation at the ICH presentation (40).

A correlation between ICH and the recurrence of bleeding, ischemic events, and other vascular complications has been reported (15).

The rate of recurrent ICH is between 4% and 7% per patient-year, very similar to that of ischemic stroke, and depends on the cause, the presence of comorbid AF, and blood pressure (41).

The site of the first hemorrhage, which is often related to the cause of the hemorrhagic stroke, is also essential (15).

ICH recurrence risk is higher in patients with lobar hemorrhage, which is usually due to CAA.

Overall, patients with bleeding diathesis and those with vascular malformations are likely to have a higher risk of ICH recurrence.

This evidence could have important implications for the approach to secondary prevention of stroke and other thromboembolic events regarding whether or not to reintroduce antithrombotic therapy (42).

Moreover, blood pressure during follow-up has a crucial role in determining the risk of recurrence. The results of the PROGRESS trial demonstrated that reducing blood pressure, even within the “normal” range, caused a lower risk of recurrent stroke, particularly in those who entered the study for hemorrhagic stroke (43).

### 3.4. ICH and AF

ICH risk in patients with AF is related to OACs treatment. In the Dijon Stroke Registry (2006–2017), AF has been reported in 97 of 444 ICH patients (21.9%).

Among them, AF was known and treated with OACs in 65 patients (14.6%) while 13 subjects (2.9%) had unrecognized AF (44). An increase in the incidence of FA was observed between 2006 and 2017, rising from 17.2 to 25.8%. Furthermore, the percentage of patients treated with OACs and the percentage of new AF significantly increased over this decade (44). In contrast, patients without AF were younger (mean age: 70 vs. 78 years) and had a lower CHA2DS2-VASc score (2.5 vs. 3.6) (45).

The CHA2DS2-VASc score is a helpful tool for risk ischemic stratification (42, 46) in patients with ICH and AF. Conversely, the HAS-BLED score has been developed to assess the bleeding risk in AF patients needing OACs (47, 48). Moreover, HAS-BLED has been taught to better predict major bleeding risk compared to other score systems such as HEMORR(2)HAGES and ATRIA (49, 50).

Nevertheless, some factors that increase ICH risk, such as old age (51, 52), a lobar location of previous ICH (53), as well as CAA (54–57), cortical superficial siderosis (58), and lobar cerebral microbleeds (59) are not contemplated in the HAS-BLED (56, 57). Notably, it has been hypothesized that the HAS-BLED score largely underestimates ICH risk in CAA patients. Indeed recurrent ICH in CAA patients has been shown to be higher than it has been expected according to the HAS-BLED score, suggesting the need for further tools for a better risk stratification (60).

### 3.5. Oral anticoagulation as a risk factor: current evidence

In patients receiving OACs, up to the 10-fold increased risk of ICH has been reported ranging from 0.25% to 1.1% (61) with a 30-to 90-day mortality rate of 40%–65% (62–65). In Denmark, from 2004 to 2017, the use of OACs among ICH patients intensified to 18% (31).

In recent decades, however, the prevalence of previous use of OAT in patients with ICH has increased by approximately 50% (66, 67). The prevalence of patients who develop ICH during OACs is slightly but significantly higher in women (67).

Remarkably, in South Limburg, in the Netherlands (68), in the period between 2007 and 2009, 25.8% of total ICH (168 of 652) have been associated with OACs use in patients on VKA. An annual incidence of 40.9 total ICH and 10.5 OAC-related ICH per 100,000 people has been reported. Conversely, 23.2% of ICH have been correlated to OACs (121 of 522): however, in this case, 70 were on VKA while 51 were on DOACs). Accordingly, in the following decade, also the annual incidence significatively decreased (reaching 7.5 per 1,000,000 person-year), despite the aging population and the augmented number of OACs users.

Data from 2,452 subjects with a previous ICH (mean age 76 years, 41% female, who were mostly on VKA) highlighted the fact that taking no antithrombotic drug increased ischemic complications without influencing the ICH rate (69).

The introduction of DOACs and changes in OACs behavior have been associated with an inferior risk of ICH compared to VKA (70).

In RCTs assessing the effect of DOACs in patients with AF, the treatment was associated with a 40%–65% risk reduction of ICH compared to warfarin (Table 1) (71–74). Although these data are robust and encouraging, new questions have arisen concerning the hematoma expansion or bleeding volumes and their prognostic impact in patients treated with DOACs or VKA.

**Table 1:** Risk of ICH in patients treated with DOACs or VKI in RCT.

RCT	DOACs	Year	ICH n° pt (%)		HR (95% CI)	
			DOACs	VKI	DOACs vs VKI	*P* value
RE-LY trial (72)	Dabigatran	2009	14 (0.2)*	45 (0.7)	0.31 (0.17–0.56)	<0.001
			12 (0.2)^§^		0.26 (0.14–0.49)	<0.001
ROCKET (73)	Rivaroxaban	2011	55 (0.8)	84 (1.2)	0.67 (0.47–0.93)	0.02
ARISTOTLE (71)	Apixaban	2011	40 (0.4)	78 (0.8)	0.51 (0.35–0.75)	<0.001
ENGAGE TIMI-48 (74)	Edoxaban	2013	49 (0.7)	90 (1.3)	0.54 (0.38–0.77)	0.001

DOACs, direct oral anticoagulant; CI, confidence interval; HR, hazard ratio; ICH, intracranial hemorrhage; RCT: a randomized controlled trial;.

* 110 mg.

§ 150 mg.

In this regard, few heterogeneous data with controversial results are available. In a multicenter study, Wilson et al. did not observe any difference between DOACs or VKA-ICH volume, with a similar 90-day rate of expansion of the initial hematoma, worse prognosis, and mortality (75). Similarly, some studies reported a comparable hematoma expansion between patients treated with DOACs or VKA (76, 77). Conversely, in an observational study that enrolled 2,245 patients with DOACs or VKA-associated ICH, Kurogi et al. reported that patients treated with DOACs were less likely to have moderate or severe impaired consciousness or need surgical hematoma removal (78). Different studies and meta-analyses of RCTs also demonstrated a reduction in in-hospital mortality in patients with ICH treated with DOACs compared to VKA (79, 80).

### 3.6. Time and modalities of OACs resumption

**Figure 2:**
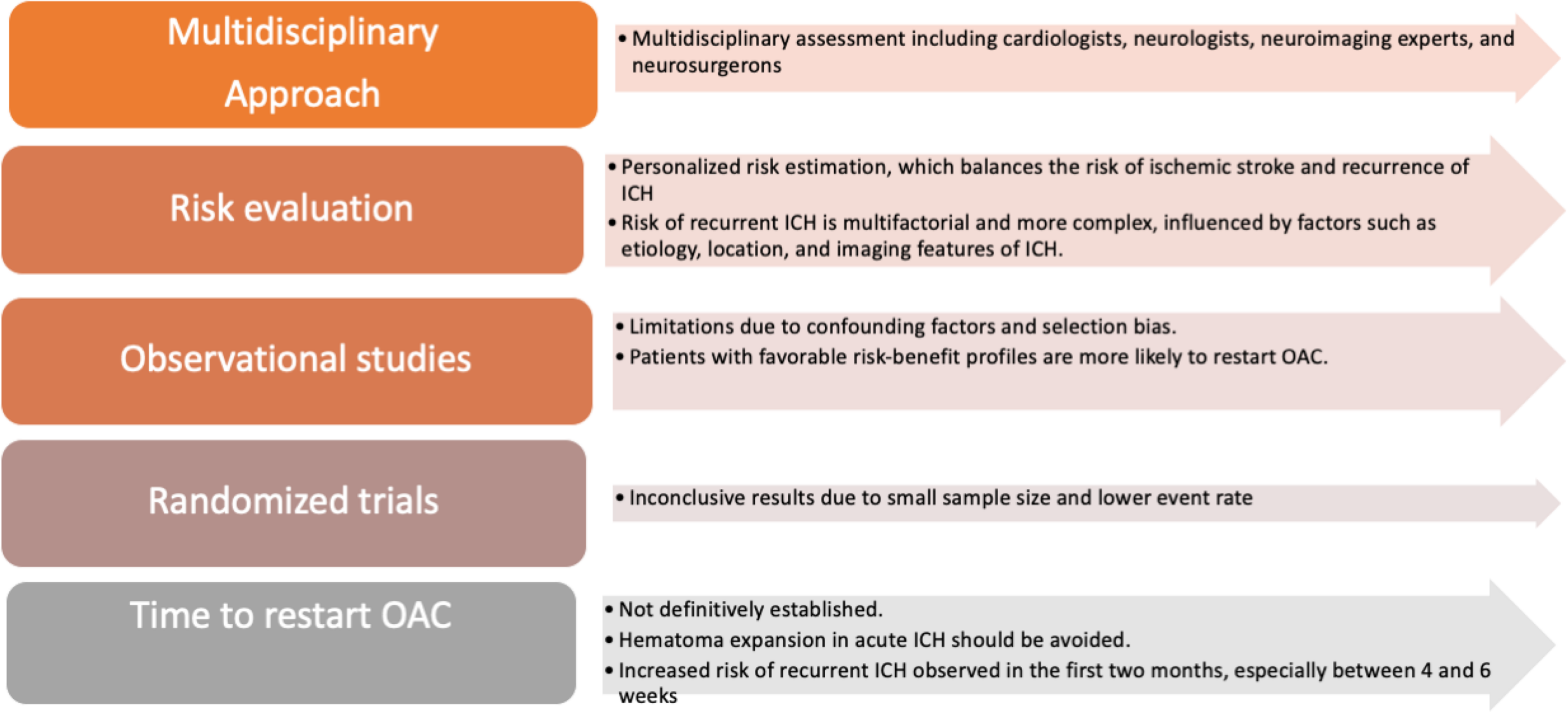
OACs resumption decision making. ICH: Intracranial Hemorrhage, OAC: Oral anticoagulant.

The resumption of OACs after intracranial bleeding represents a crucial clinical conundrum (Figure 2). Although a great risk of ischemic stroke in patients with AF and a history of ICH has been confirmed, this population has been substantially excluded from phase 3 RCTs of OACs for stroke prevention in spite of the higher ischemic risk (81). Thus, most available data derive from observational studies (3, 8, 9). According to the latest guidelines (82), the clinical choice of restarting anticoagulation should be made after a multiparametric evaluation taking into account not only the cardiologic point of view but also neurological, neuroimaging, and neurosurgery aspects (82). Furthermore, a personalized risk estimation balancing the recurrence of ICH and ischemic stroke risk should mostly influence what strategy to adopt (82). Though the ischemic risk profile could certainly be evaluated with a CHA_2_DS_2_-VASc score, the risk of recurrent ICH is multifactorial and more complex to be estimated because of the high variability in its incidence has been reported ranging from 1.3% to 7.4% (3). Etiology, location (lobar ICH at higher risk than non-lobar), and imaging features of ICH can help predict the risk of recurrence (Table 2).

**Table 2:** Principal factors associated with the risk of recurrence of intracranial hemorrhage (ICH).

Factor	Lower risk of recurrence	Higher risk of recurrence
Type of ICH	Subdural ICH, Epidural ICH	Subarachnoid ICH, Lobar ICH
Cause of ICH	Traumatic	Spontaneous
Size of ICH	Mild (i.e. volume)< 30 ml	Moderate to severe
Presence of cerebral microbleedings	No	Yes
Amyloid angiopathy	No	Yes

ICH: intracranial hemorrhage.

Etiology, location, and imaging features of ICH are important factors in predicting the risk of recurrence Spontaneous bleedings are more likely to recur than traumatic ones, especially if cerebral microbleeds are shown by imaging. Indeed, in patients with a traumatic ICH, the restarting of OACs is likely to be related to a lower incidence of ischemic stroke and mortality rate (83). Notably, in these subjects, a higher recurrence of ICH has also not been reported (83). On the contrary, a relationship between anticoagulation strategy and recurrent ICH has been described in patients with AF after a non-traumatic ICH. Remarkably, the cerebral microbleeds (CMBs) neuroimaging finding is likely to be more strictly associated with a greater recurrence of ICH (84).

In the absence of antithrombotic therapies, an increased incidence of ischemic stroke compared to the recurrence of ICH has been shown in 2,452 AF patients with a previous ICH (mean age 76 years, 41% female, VKA was the most used OACs) (69). Notably, a significant reduction of thromboembolic risk in patients who have restarted OACs without raising the recurrence of ICH has been demonstrated (85).

However, these observational studies are burdened by limitations due to confounding factors and selection bias, as patients with favorable risk-benefit profiles were more likely to restart OACs (86). Data from RCTs are therefore warranted, and several studies are ongoing. Recently, data from the first two RCTs on this topic were published. Neither trial observed significant differences between OACs and no therapy for ischemic stroke or ICH. Although results were inconclusive due to the small size of the enrolled population and the lower event rate than expected, a trend favoring OACs resumption was found (87, 88).

It has not been definitively well established how much time from an ICH is needed to restart OACs (15). Hematoma expansion, expected in acute ICH, is aggravated by anticoagulation. Therefore, in the acute phase of ICH (<24–48 h), anticoagulant treatment should be avoided, and strategies to reverse anticoagulation should be considered (89, 90). An increased risk of recurrent ICH has been observed in the first two months, especially between 4 and six weeks after the index event (91). A wide range of optimal time range from 72 h (92) to 10–30 weeks (91) has been reported. A recent retrospective study performed in Korea showed that waiting 6–8 weeks after ICH for restarting anticoagulants was the safest choice in terms of lower risk of all-cause mortality while resuming anticoagulants after 4–6 weeks after ICH was riskier for bleedings (93).

Conversely, a 70-day waiting period to resume the anticoagulant has been shown to be likely to reduce the recurrence of events; therefore restarting at least after 28 days has been suggested (94).

Accordingly, on the basis of the Swedish registry data involving 2,619 adults, a waiting period of 7–8 weeks for restarting OACs in order to balance the observed risk of ischemic and hemorrhagic complications has been recommended (42). Moreover, restarting OACs six weeks after ICH would not have been correlated to an increased risk of intracranial bleeding over one year of follow-up (69). The majority of available data derived from patients treated with VKA and shorter times could be hypothesized with DOACs. Indeed, current guidelines recommend DOACs over VKA in DOACs-eligible patients (82). Overall, available data support a resumption of OACs at 7–8 weeks after ICH (42).

### 3.7. Resumption in specific cases: patients with lobar ICH

ICH location is essential in deciding whether and when to resume OACs (Table 3).

**Table 3:** OACs resumption modalities according to ICH subtype.

ICH-Clinical Features	Physiolopatology	Risk of recurrence	OACs resumption decision-making	OACs resumption
Lobar ICH	Strictly related to CAA, with the characteristic presence of amyloid-*β*, micro-hemorrhages, and fragile vessel structure	Very high risk of recurrence. A genetic apolipoprotein E association in CAA-related lobar ICH has been described as a cause of the recurrence of ICH.	OACs resumption decision-making is challenging and it should be followed a comprehensive diagnostic work-up including a magnetic resonance, in order to identify microbleeds and neuroimaging markers for increased hemorrhagic risk such as cSS or cSAH.	– f CAA is suspected and in the presence of a microbleeds burden > 5, OACs should be avoided, and alternative strategies should be considered – If CAA is not probable and a microbleeds burden < 5 occurs, OAC should be restarted between 4 and 8 weeks after ICH should be restarted according to the patients's individual thromboembolic/hemorrhagic risk evaluation. DOACs over VKA should be preferred
Non-lobar ICH	Hypertensive vasculopathy	Lower than lobar location	A secondary ICH etiology should be excluded.	OACs regimen between 4 and 8 weeks after ICH should be restarted according to the patients’ individual thromboembolic/hemorrhagic risk evaluation. DOACs over VKA should be preferred
ICH in patients with mechanical heart valves	Altered, non-therapeutic coagulation		Challenging due to the high risk of ischemic events, and VKA are the only option.	OACs should not be restarted earlier than six days after the initial ICH. In patients with high thrombotic risk (concomitant AF, mitral position, or older prosthesis types) OACs may be restarted after one week. OACs Resumption after 13 days from ICH is generally considered safe.

ICH: intracranial hemorrhage; CCA: cerebral amyloid angiopathy; cSS; cortical superficial siderosis; cSAH: cortical or convexity subarachnoid hemorrhage; OACs: oral anticoagulants.

It has been well assessed that lobar ICH is associated with CAA; moreover, it is likely to recur more than other ICH subtypes (3, 95). On this account, according to the latest American guidelines (96), OAT after a lobar ICH should be avoided (97). Therefore, in patients with lobar ICH, it is essential to assess the possible presence of CAA using the modified Boston Criteria (98). MRI should also be integrated into the diagnostic workup before considering OACs resumption, as it could help in estimating the risk of ICH recurrence. Remarkably, the cerebral microbleeds (CMB) burden detected by iron-sensitive imaging is likely to be related not only to the occurrence of ICH but also to the recurrence after ICH in patients treated with OACs (99). Moreover, neuroimaging should be a helpful tool in recognizing particular conditions at higher hemorrhagic risk as acute convexity subarachnoid hemorrhage (cSAH) and cortical superficial siderosis (cSS) (99). The association of CAA and cSAH has been shown to be related to an ICH rate of 19% per patient-year (100). On the contrary, very few data are available about the risk of recurrent ICH in patients with CAA on OACs (101).

A sub-group analysis among 190 patients with probable or possible CAA from a more extensive meta-analysis showed a lower mortality rate and better functional parameters in patients who had restarted OACs, although this data was not sufficient to confirm a role of OACs in the outcomes (102). In conclusion, in subjects with lobar-ICH, an MRI evaluation of the presence of microbleeds, cSS or cSAH might lead to the decision to restart OACs or to adopt other strategies (i.e., LAA closure) (86).

### 3.8. Resumption in specific cases: patients with mechanical valves

In the presence of mechanical heart valves (MHV), OACs resumption is particularly challenging (Table 3). Firstly, the risk of ischemic events is particularly high in this population reaching 4% patient-years in the absence of OACs and reducing to 1% patient-years with anticoagulant treatment (103). According to the type and seat of the prosthesis, especially if AF coexists, the thromboembolic risk might boost (104). Secondly, in light of the fact that DOACs are not indicated in these patients, VKA is considered the only pharmacological opportunity.

For patients with MHV and a concomitant ICH, introducing the heparin anticoagulation strategy three days after the event and switching to VKA seven days later has been considered safe (105). Recently German multicentric RETRACE study involving 2,504 patients on OACs who survived ICH patients (166 with MHV) showed that restarting anticoagulation (heparins or VKA) within 14 days after an ICH has been associated with a significantly higher occurrence of major and intracranial bleedings (106). Conversely, OACs resumption seems to be safe after two weeks (106). In contrast, it could be reasonable to wait at least one week in patients in which thromboembolic risk is particularly high due to a concomitant AF, mitral prosthesis, or history of embolism) only to restart OACs (106).

### 3.9. Further caution measures for restarting DOACs in patients with previous ICH

When the decision to restart DOACs has been made, further preventive measures should be adopted in order to improve the safety of patients considered eligible.

On this matter, it has been claimed that the use of P-glycoprotein (P-gp) and cytochrome P450 (CYP) 3A4 inducers or inhibitors in patients concomitantly on DOACs, must be carefully taken into account considering that they could have an impact on anticoagulation. Notably, the assessment of potential interactions in ICH survivors on DOACs is nowhere as easy as it is in other patients and a more accurate examination ought to be done in order to avoid potential unexpected events.

**Figure 3:**
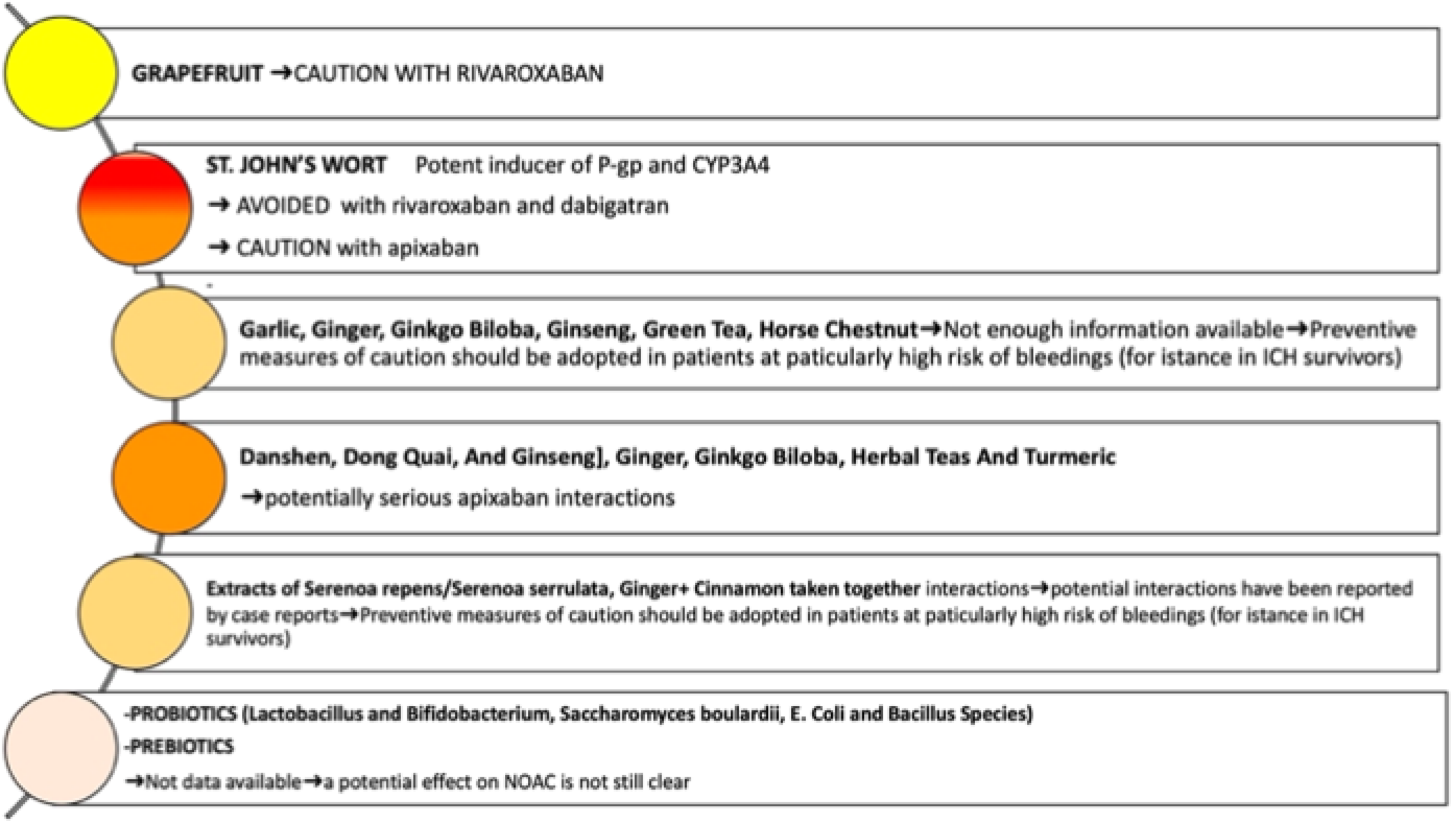
Potential effects of herbal medicines, probiotics and prebiotics on DOACs. Further preventive measures in patients with previous ICH should be adopted in order to improve the safety of patients considered DOACs eligible. Caution is needed in assessing potential interactions DOACs with other substances in ICH survivors to prevent them from potential adverse effects. The use of herbal products, food supplements, probiotics, and prebiotics, should be accurately evaluated.

Sánchez-Fuentes et al. (107) recently described the potential effects of herbal medicines, dietary supplements, and foods highlighting their potential interactions with DOACs. The findings of their interesting research (107) can be applied in clinical practice even in these patients who are certainly more complex in consideration of their clinical history. In addition, due to the growing interest in herbal products, probiotics, and prebiotics, and their widespread use, concomitant use with DOACs is likely to occur. The potential interactions are shown in the Figure 3.

Moreover, the potential effects of probiotics and prebiotics on anticoagulation strategy have also been examined (107). Although several beneficial effects have been confirmed (108, 109), their influence on gut microbiota composition is expected to interact with VKA. Consequently, an increase in the anticoagulant effect has been supposed. However, their potential interaction with DOACs is still not well investigated.

Overall, these results (107) suggest that it is worth improving the awareness of the potential impact of herbal medicines, dietary supplements, probiotics, and prebiotics in patients on DOACs and ICH survivors should be carefully evaluated.

### 3.10. Non-pharmacologic options (percutaneous left atrial appendage closure)

It has been assessed that approximately ninety percent of thrombi occur within the LAA in patients with AF (110). The discovery of LAA as the anatomical site with a higher probability of thrombus formation in AF patients led to non-pharmacological approaches. The mechanical occlusion of LAA aims to prevent embolization of any possible thrombus formed inside. Therefore, a percutaneous technique to obliterate LAA was developed. LAA closure (LAAC) is routinely performed *via* a venous transcatheter femoral access. A self-expanding device with a polymer membrane is delivered in the LAA throughout a trans-septal puncture in order to exclude the LAA cavity from the rest of the atrium, thereby obliterating the site that is the nidus for thrombus formation (111).

A meta-analysis of RCT on LAAC vs. DOACs (112) (1,516 patients from PROTECT AF (113, 114), PREVAIL (10, 115), and PRAGUE-17 (116); LAAC 933, OAC 583, in OAC group 65% warfarin, 35% DOACs), after a mean follow-up of 38.7 ± 17.2 months, showed that ischemic stroke incidence was similar in the two population.

However, OACs was associated with significantly more hemorrhagic strokes, cardiovascular death, and all-cause mortality.

A significant difference in major bleeding has not been reported, though non-procedure-related major bleeding favored LAAC.

Patients with a prior history of ICH have not been excluded from PROTECT (114) and PREVAIL (115) trials whose results allowed the introduction of LAAC in clinical practice.

Currently, recommendations for LAAC are not yet defined (Table 4).

**Table 4:** Recommendation for left atrial appendage occlusion according to international guidelines.

Guidelines	Recommendation	Grade of recommendation and level of evidence
ACCP 2018 (117)	LAA occlusion has been suggested in AF patients at high risk of ischemic stroke who have absolute contraindications for OACs	Weak recommendation, low quality of evidence
Taiwan heart rhythm society 2018 (118)	Percutaneous LAA closure may be considered in patients with very high stroke risk and contraindicated for long-term OACs	No grading
Cardiac society of Australia and New Zealand 2018 (119)	LAA occlusion may be considered for stroke prevention in patients with N-VAF at moderate to high risk of stroke and with contraindication to oral anticoagulation therapy	Strong recommendation, weak quality of evidence
AHA/ACC/HRS 2019 (120)	Percutaneous LAA occlusion may be considered in patients with AF at increased risk of stroke who have contraindications to long-term anticoagulation	COR: IIb, LOE: B
CCS 2020 (121)	Percutaneous LAAO should be considered for stroke prevention in patients with NVAF who are at moderate to high risk of stroke and have an absolute contraindication to OACs	Weak recommendation, low quality of evidence
ESC 2020 (82)	LAA occlusion may be considered in patients with AF and contraindications for long-term anticoagulant treatment (e.g intracranial bleeding without a reversible cause)	COR IIb, LOE B

AF = atrial fibrillation; OACs = oral anticoagulation; LAA = left atrial appendix; LAAO = left atrial appendix occlusion; NVAF = non valvular atrial fibrillation; COR = class of recommendations; LOE = level of evidence.

Table 4 indication for left atrial appendix closure in the current guidelines.

Despite the lack of RCTs on LAAC in patients with previous ICH, there is every likelihood that LAA occlusion could be a beneficial option in these patients (122–127). LAAC could represent the only alternative in patients with an absolute contraindication to OACs.

A recent meta-analysis (11) of 7 retrospective studies enrolling 407 high-risk patients with a history of ICH who underwent LAAC (mean CHA2DS2VASC and HAS-BLED scores were respectively 4.8 ± 1.5 and 4 ± 1) showed promising results: an acute procedure success rate in 98.5% of the patients and a low rate of periprocedural complications (pericardial effusion 0.17%, device embolization 0.1%, device-related thrombosis 0.03%, major bleeding 0.02%, recurrent ICH 0% (95% CI: 0–0.56). At long-term follow-up, major bleeding and recurrent ICH (0.25% and 0.05%, respectively) occurred, resulting inferior to what would be expected on account of the high HAS-BLED score. Accordingly, also ischemic stroke incidence (0.54%) turned out to be lower than what would have been expected in the absence of OACs for a CHA2DS2VASC >4 (11). The anti-thrombotic regimen following LAAC was variable across countries. Combination therapy with warfarin and acetylsalicylic acid (ASA) (81–325 daily) has been used in the main RCTs (10, 113–115, 128, 129) for one and half months after the procedure until the 45 days transoesophageal echocardiography (TOE) follow-up. Provided that a residual shunt >5 mm and device surface thrombi (DST) did not occur, OACs was interrupted, and dual antiplatelet therapy (DAPT) regimen (ASA and Clopidogrel 75 mg daily) was prolonged for six months, followed by a lifelong single antiplatelet therapy (SAPT) approach (ASA 325 mg/day). An annual bleeding rate of 1.2%, 0.6%, and 3.1% at 45 days, six months, and five years FU has been reported, respectively (130, 131).

In 1,000 recipients who had undergone patent foramen ovale (PFO) or atrial septal defect (ASD) closure, DST occurred in 15%, 30%, and 55% of patients receiving warfarin, SAPT (ASA alone), and DAPT (ASA and clopidogrel), respectively (132).

Furthermore, in another analysis, the 99% of 143 subjects who successfully had LAA occlusion, received DAPT (clopidogrel and ASA) for 30–90 days, followed by SAPT (ASA only) for  five months, showing a low thrombogenicity of AMPLAZER devices (133). In the Amplatzer Amulet device registry (134), 1,088 subjects, 71.7% with a history of major bleeding and 82.8% with contraindications to OACs, underwent LAAO closure. TOE was performed 30–90 days after closure, showing a complete procedure in 98.4% of cases and 1.6% of device-related thrombus (DRT). At discharge, 57.7%, 22.4%, and 11.2% received DAPT, SAPT, and OACs, respectively. Notably, the ischemic stroke was reduced by 67% compared to what would have been expected based on the CHA2DS2-VASc score, reporting an annual event rate of 2.2%, showing safe results in more than 80% of patients received exclusively APT (134). A DRT incidence rate of 2.3% has been reported in a metanalysis of 2,855 patients successfully treated with low-molecular-weight heparin for two weeks (135).

However, what makes the antithrombotic strategy challenge most, is that, in the real world, the majority of patients who were referred to LAAO closure have a history of bleeding and are considered unsuitable for VKA or DOACs (117, 136–138). Moreover, patients with contraindications to OAC sor DOACs have been excluded from RCTs. Therefore, due to the lack of RCTs, thromboembolic prevention is particularly complex in this subset of patients so that physicians, for fear of bleeding, usually consider minimal regimens as a 2-week DAPT, followed by SAPT, avoid prescribing OAC (139). A few preliminary data suggested that a regimen based on a low dose DOACs could be used instead of a full dose in very high-risk patients, showing the same thromboembolic protection after LAAC; however, randomized data are missing (140–144). In some extreme cases of high-bleeding risk patients, a single antiplatelet therapy or even no antithrombotic treatment was used after LAAC. A few small studies have evaluated the safety of SAPT following LAAC, and the result is not always concordant (142, 145–148).

Another approach consists of continuing DAPT until a six-month TOE follow-up and then deciding accordingly (139). Generally, if TOE excludes residual shunt >5 mm jet and device surface thrombi (DST), SAPT is continued (139). However, ASA 75–325 mg/day should be continued long-term (149), although it is frequent that antithrombotic agents are stopped within the first 12 months in patients without particular indications (139).

DAPT strategy after PFO closure seems to be effective in avoiding thrombus development despite bleeding complications (131, 150–153). However, the optimal DAPT duration remains largely debated.

After Watchman implantation, two main antithrombotic strategies have been proposed.

On one hand, according to bleeding risk, OACs should be prescribed for 45 days, followed by clopidogrel for six months, in low-bleeding-risk patients, whereas it should be avoided in those at high-risk (149). On the other hand, in patients with contraindications for OACs, DAPT (clopidogrel and ASA) may be continued for 1 to 6 months after the procedure (149). Furthermore, after AMPLATZER or Amulet implantation, DAPT (clopidogrel and aspirin) may be prescribed for 1 to 6 months after the procedure (149). A tailored approach may be adopted, including SAPT (ASA or clopidogrel) for a limited period, considering a team-based evaluation (149).

Another methodology that could be useful in particular cases is the epicardial ligation of the LAA for reducing the anticoagulant treatment both during and after the procedure, although a non-negligible incidence of residual leaks has been reported (154–157). The best approach to address this problem could be to discuss the issue in a multidisciplinary “stroke team” and tailor the post-procedural therapy based on patient characteristics.

Recent guidelines have highlighted the importance of a multidisciplinary “stroke team” formed ideally by neurologists, cardiologists, neuroradiologists, neurosurgeons, patients, and their families, which should be in charge of the therapeutic decision for each patient, tailoring the most appropriate therapy to patient characteristic. We think that nowadays, due to the lack of RCTs data, the routine reference to this team could represent the best approach to cope with complex cases.

One of the main issues of LAAC implementation is that there is no high-quality data on the duration and the type of therapy OAT after LAAC implantation, and there is a wide variety of treatments across different centers (150). Usually, OACs are used for a short period after LAAC. However, which OACs should be used and the optimal duration of this treatment is not established, and there is a high variation among registries (150). In the subset of patients with an absolute contraindication to OACs, dual antiplatelets with aspirin and clopidogrel could be used (150). The safety of this approach is currently unclear. In a meta-analysis of 83 observational studies (150), enrolling 12,326 patients compared short-term oral anticoagulation vs. dual antiplatelet, reported no difference in bleeding, stroke, device-related thrombus, and all-cause mortality. Of note, this meta-analysis does not report the percentage of patients with a history of ICH in the studies. The best approach to address this problem could be to discuss the issue in a multidisciplinary “stroke team” and tailor the post-procedural therapy based on patient characteristics.

In conclusion, LAAC is a safe alternative to reduce ischemic stroke in OAC-ineligible patients, in which this procedure is the only feasible therapeutic option.

Future clinical trials focused on choosing optimal anticoagulation strategies after ICH would be helpful for improving the therapeutic strategy in these patients.

### 3.11. Guidelines indications

Recommendations for restarting OACs have been summarized in Table 5.

**Table 5:** When to restart OAC according to international guidelines.

Guidelines	Year	Class of Recommendation (Level of evidence)	Resumption Recommendations
ACC Expert Consensus (158)	2017	–	Multidisciplinary approach for high-risk cases, 4-week waiting for DOACs
CHEST Guideline (117)	2018	Ungraded consensus-based statement	From 48 h to 4 weeks based on individual risk/benefit evaluation. DOAC preferred. LAA occlusion for high recurrent ICH risk
ESO-Karolinska Stroke Update (159)	2019	C	4–8 week waiting, individual decision-making, DOACs for NVAF
ESC Guidelines (82)	2020	IIa (C) IIb (B)	OACs re-initiation (2–4 weeks), after careful evaluation of individual risks and benefits, DOACs preferred. LAA occlusion for high recurrent ICH risk.
EHRA Practical Guide (160)	2021	–	4–8 week waiting after multidisciplinary team assessment, consider no anticoagulation or LAAO

ACC, American College of Cardiology; CHEST, American College of Chest Physicians; DOACs, Direct oral anticoagulants; NVAF, Non-valvular Atrial Fibrillation; LAAO, Left Atrial Appendage Occlusion; EHRA, European Heart Rhythm Association.

According to 2018 consensus statements and recommendations from the European Stroke Organisation (ESO) 2018 (159).

It would be advisable to restart OACs in selected ICH patients rather than not using OACs in view of better outcomes not accompanied by an increased ICH recurrence rate for subjects with AF who survived an ICH (Grade C). Therefore DOACs could be considered safer compared to VKA in these patients (Grade C).

The re-introduction of OACs between the first four to eight weeks from index ICH is considered quite reasonable (Grade C) (159). A tailored decision-making on OACs after ICH should also take into account factors such as BP control, age, where ICH is located, and its dimension; in addition neuroimaging findings such as leukoaraiosis, cortical superficial siderosis, CAA should be considered (Grade C) OACs between the first four to eight weeks from index ICH seems to be safe (Grade C) (159).

According to the 2018 CHEST guidelines (117), anticoagulation with a DOACs after acute spontaneous ICH must be carefully evaluated, considering the risks and benefits. Moreover, for those selected patients at high risk of recurrent ICH, as in the case of a concomitant diagnosed or suspected CAA, LAA occlusion is suggested (117).

According to the 2019 American Heart Association and American Stroke Association (AHA/ASA) guidelines (161), it is reasonable to initiate OACs between 4 and 14 days after the onset of neurological symptoms for most patients with an acute ischemic stroke in the setting of AF.

According to the 2020 European Society of Cardiology (ESC) guidelines (161), the choice of restarting OAC in AF patients at high thromboembolic risk should be based on a multidisciplinary approach balancing potential advantages and disadvantages. DOACs should also be preferred compared to VKA (Class II a).

Conversely, a recent high-risk bleeding event such as ICH (within two weeks) is considered an absolute contraindication to OACs (161). Moreover, in AF patients at very high risk of recurrent ICH, LAA occlusion may be considered (161). In more detail, LAA occlusion may be a therapeutic option in patients with AF and intracranial bleeding without a reversible cause (Class recommendation IIb, level of evidence B) (161).

According to the 2021 European Heart Rhythm Association (EHRA) guidelines, the best timing to restart anticoagulation is after 4–8 weeks after a multidisciplinary assessment, and if considered not suitable, LAA occlusion is strongly recommended (160).

## 4. Conclusion

Robust data available on the timing and modalities of OACs resumption after a major bleeding event, such as an ICH, are lacking. Patients with a history of recent ICH were excluded from RCTs on stroke prevention in AF, so the recommendations of current guidelines are weak.

For fear of ICH, a potentially lethal adverse effect of anticoagulation regimen, physicians are commonly reluctant to reinitiate OACs in AF patients who survived an ICH, even in patients with a high estimated risk of AF-related ischemic stroke. The decision-making should be based on a multi-specialist approach. A careful balance of the advantages and disadvantages of restarting OAC must be considered.

## Data Availability

The original contributions presented in the study are included in the article/Supplementary Material, further inquiries can be directed to the corresponding author/s.

## Group Members of Quality Working Group ANMCO, Cardiac Chronic Diseases ANMCO, and Arrhythmia Working Group ANMCO

Members of Management and Quality Group Working Group, ANMCO Fabiana Lucà, GOM Hospital, Bianchi Melacrino Morelli, Reggio Calabria, Italy (Chairperson); Simona Giubilato, Cannizzaro Hospital, Catania, Italy; Giorgio Caretta, S. Andrea Hospital, La Spezia, Italy; Stefano Cornara, Levante, San Paolo, Hospital, Savona, Italy; Irene Di Matteo, ASST, Niguarda Hospital, Milano, Italy.

Anna Members of Arrhythmias Workin Group ANMCO, Raimondo Calvanese, Ospedale del Mare, Napoli, Italy (Chairperson); Carlo Pignalberi San Filippo Neri Hospital; Roma, Italy; Francesco Borrello, Civile Pugliese Ciaccio Hospital, Catanzaro, Italy; Amir Kol, S.Camillo de Lellis Hospital, Rieti, Italy; Martina Nesti, San Donato Hospital, Pasquale Vergara, IRCCS San Raffaele Hospital, Milano, Italy; Manuel Antonio Conti, Civile G. Mazzini Hospital, Teramo, Italy; Federico Migliore, Università degli Studi di Padova, Italy; Pietro Rossi, Fatebenefratelli Hospital, Roma, Italy.

Members of Cardiac Chronic Diseases Working Group, ANMCO, Maurizio Giuseppe Abrignani, Paolo Borsellino Hospital, ASP Trapani, Marsala, Italy (Chairperson); Pier Luigi Temporelli, Istituti Clinici Scientifici Maugeri IRCCS, Gattico-Veruno, Novara, Italy; Giulio Binaghi, ARNAS G. Brotzu Hospital, Cagliari, Italy; Arturo Cesaro.
